# Has GBL Replaced GHB in Recreational Settings?

**DOI:** 10.2478/aiht-2020-71-3424

**Published:** 2020-06-29

**Authors:** Anastasio Tini, Alessandro Del Rio

**Affiliations:** 1 Marche Polytechnic University, Department of Excellence SBSP, Section of Legal Medicine, Unit of Forensic Toxicology, Ancona, Italy; 2 Sapienza University of Rome, Department of Anatomical, Histological, Forensic, and Orthopaedic Sciences, Rome, Italy

Dear Editor,

We have read with great interest the review article by Marinelli et al. (1) entitled “Gamma-hydroxybutyrate abuse: pharmacology and poisoning and withdrawal management”. This review focuses on updated knowledge about GHB pharmacokinetics and pharmacodynamics, acute poisoning, and clinical features of GHB withdrawal syndrome, its diagnosis, and medical treatment.

In addition, the authors give special emphasis to “new frontiers in GHB” taking into consideration the emerging role of -butyrolactone (GBL) and 1,4-butanediol (1,4-BD).

While we agree with the considerations raised by Marinelli et al. (1), we wish to further discuss the role of GBL, which has completely replaced the use of GHB as white powder in recreational settings but also as a date rape drug (2, 3, 4). Besides GBL, GHB can be obtained in its sodium salt form (sodium oxybate), which is currently marketed for the treatment of alcohol withdrawal syndrome (Alcover?) and narcolepsy with cataplexy (Xyrem?) (5, 6).

Marinelli et al. (1) stated that 1 mL of pure GBL contains about 1.6 g of GHB, but GBL significantly varies in the degree of purity. Analysing 30 illicit preparations generally sold as “G”, Busardò et al. (2) highlighted the presence of GBL in all of them at a mean concentration of 760.7±91.46 mg/mL and a wide range of 588.5–899.3 mg/mL. The consequence of this variability is that users cannot base the dosage on volume and therefore run a higher risk of overdose than with GHB.

Presently, the main concern associated with GBL abuse is the lack of data about its actual distribution and its rapid conversion into GHB after ingestion. Moreover, in cases of acute poisoning and fatalities only GHB is looked for in biological samples and symptoms are univocally attributed to GHB (2, 7, 8). This calls for highly sensitive analytical methods that would be able to detect not only GHB but also its precursors (and metabolites) in conventional and non-conventional biological matrices. Speaking of the latter, hair seems to have a great potential for this type of analysis (9). Considering GBL’s availability in certain contexts such as in homosexual clubs and chemsex parties, it is fundamental to develop analytical strategies able to detect not only GHB/GBL but also other associated classes of substances, mainly synthetic cathinones such as mephedrone (10, 11).

In conclusion, we firmly believe that GBL currently represents a growing public health issue, since the substance is relatively cheaper and easier to obtain than GHB (12, 13). Improvement and implementation of laws and policies to place GBL under control are needed to limit its distribution, considerable abuse, and associated health and dependence risks.
